# Public Mood and Consumption Choices: Evidence from Sales of Sony Cameras on Taobao

**DOI:** 10.1371/journal.pone.0123129

**Published:** 2015-04-22

**Authors:** Qingguo Ma, Wuke Zhang

**Affiliations:** 1 School of management, Zhejiang University, Hangzhou, China; 2 Neuromanagment Lab, Zhejiang University, Hangzhou, China; Wenzhou University, CHINA

## Abstract

Previous researchers have tried to predict social and economic phenomena with indicators of public mood, which were extracted from online data. This method has been proved to be feasible in many areas such as financial markets, economic operations and even national suicide numbers. However, few previous researches have examined the relationship between public mood and consumption choices at society level. The present study paid attention to the “Diaoyu Island” event, and extracted Chinese public mood data toward Japan from Sina MicroBlog (the biggest social media in China), which demonstrated a significant cross-correlation between the public mood variable and sales of Sony cameras on Taobao (the biggest Chinese e-business company). Afterwards, several candidate predictors of sales were examined and finally three significant stepwise regression models were obtained. Results of models estimation showed that significance (F-statistics), R-square and predictive accuracy (MAPE) all improved due to inclusion of public mood variable. These results indicate that public mood is significantly associated with consumption choices and may be of value in sales forecasting for particular products.

## Introduction

Traditionally, behavioral economics emphasized the effect of emotion on individual behavior and decision-making. When there are enough people in the same mood, it may have considerate influence on related events. For instance, public mood has been shown to have an influence on presidential election [1], economic indexes [2], fluctuation of financial markets [3–5] and even the number of national suicides [6]. Moreover, public mood information is also valuable in predicting related events and indexes [7–11]. However, few prior studies have examined the relationship between public mood and consuming choices at society level, let alone applying public mood information to make a prediction.

In previous studies, public mood information was extracted from news titles, survey data [5], searching data from Google [2] as well as social media [1, 3–6, 10]. Among these sources, data from social media has been widely applied in forecasting financial indexes [3, 4], economic indexes [2] and suicide numbers [6]. Moreover, compared with offline data, social media data has been demonstrated to be more accurate in predicting financial indexes, which also has a greater lead time [5].

The territorial dispute between China and Japan has been around for a long time and the conflict was more intense in July 2012 because Japanese government announced the ownership of Diaoyu Island at that time. This led to a sharp fluctuation of Chinese public mood and subsequently changed Chinese consumption choices of Japanese products.

With the rapid development of IT and social media in China, to extract public mood information from social media becomes available, and makes it possible to study the relation between public mood and consumption choices. Because Sony camera is a familiar Japanese product and is widely consumed in China, it was adopted to study the influence of Chinese public mood on the sales of Japanese products during the period of the “Diaoyu Island” event. The key points of this study were to extract public mood data from social media and to explore its relationship with the sales of Japanese products. This relationship would contribute to the building of forecasting models with public mood as independent variable and sales data as dependent variable.

## Materials and Methods

### Sales data

Without official authorization, it would be impossible to collect complete sales data (daily or weekly) of Sony camera in China. Considering that Taobao (www.taobao.com) is the biggest Chinese e-business company with more than 3.7 hundred million members and tens of millions daily deals, we chose Taobao as sales data resource. In 2012/10/10, we collected daily sales of Sony camera (S_t_) from 2012/8/1 to 2012/10/8 on shu.taobao.com. These data can be seen in S1 Dataset.

### Social Media Data

Sina Microblog is one of the leading social media in China, users of which include sport and movie stars, enterprise managers, media practitioners, government officials and other people from nearly all industries. Thus, Sina Microblog was chosen as the data source of public mood information.

As has been mentioned above, Japanese government announced the ownership of Diaoyu Island in July, 2012, which led to a fierce diplomacy conflict between China and Japan. In the present study, we defined the measurement: daily original blogs (B_t_) as the number of daily original blogs simultaneously mentioning the Chinese words “Diaoyu Island”, “territory”, “sovereignty” and at least one of the following terms: “boycott of Japanese goods”, “defending Diaoyu Islands”, “defending sovereignty”, “defending territory”, “fighting for sovereignty”, “fighting for territory”, “protesting against the Japanese government”, “disdaining Japan” and “disdaining Japanese government”. The daily original blogs (B_t_) might reflect negative public mood of Chinese toward Japan and Japanese products. A similar method has been adopted in previous studies. [5, 6] For example, one study used the Tweet volumes of financial search terms (TV-FST) as negative mood variable of financial market. [5] And another one used the daily document frequency mentioning particular words as negative public mood variable to forecast national suicide numbers. [6] All the media data used in this study can be collected from www.weibo.com, and we collected these data in 2012/10/10. These data can be seen in S1 Dataset.

### Ethics Statement

This study collected existing data that were publicly available on the Internet. No individual and personal details were identified. Therefore, ethics approval was deemed unnecessary.

### Statistical analysis

Cross-correlation analysis is the basic method of forecasting a time series with another one and cross-correlation coefficients can be very helpful in building prediction models. However, this method is not always working. A better method is to find suitable functions to change time series and let the changed time series to be expectedly significantly cross-correlated. With the new cross-correlation, forecasting models can be built including autocorrelation of dependent variables. The key point in exploring correlational relationship in big data is also finding the appropriate functions to change variables and make them significantly correlated.

Based on these rules, cross–correlation analysis between daily original blogs (B_t_) and daily sales of Sony camera (S_t_) was conducted at first place in order to explore direct cross–correlation. Afterwards, we explored appropriate functions to change the two time series data to get a better cross-correlation. After that, partial autocorrelation analysis of daily sale data of Sony camera (S_t_) was conducted to study the effect of advance sales on later ones. Finally, three stepwise regression models were built with the two correlation coefficients (cross-correlation and partial autocorrelation) and were evaluated according to Mean Absolute Percentage Error (MAPE). All statistical analyses including variable selection and models construction were performed using SPSS19.0.

## Result

### Trends of daily original blogs (B_t_) and daily sales of Sony camera (S_t_) with cross-correlation analysis

**Fig 1:**
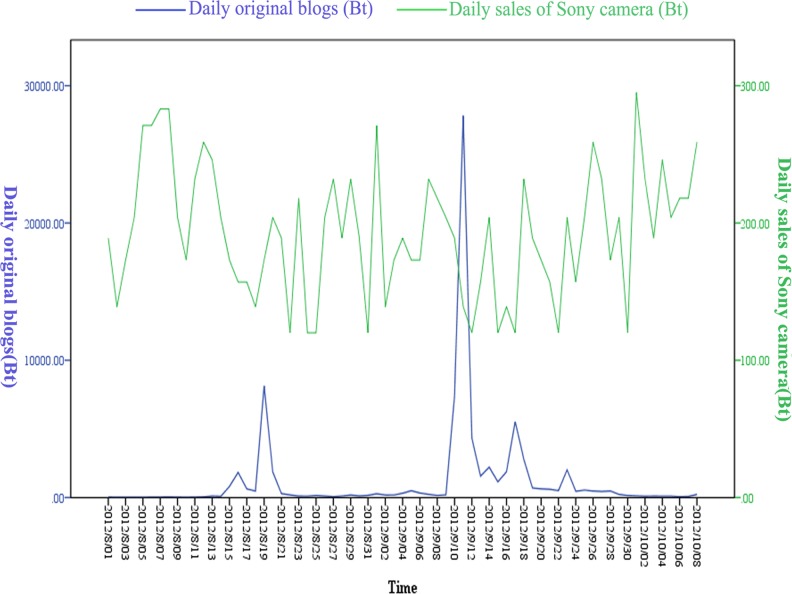
Trends of daily original blogs (B_t_) and daily sales of Sony camera (S_t_).

Over the 70-days period of this study, both daily original blogs (B_t_) and daily sales of Sony camera (S_t_) experienced obvious fluctuations (Fig 1). Compared with S_t_, B_t_ experienced fiercer variation, booming from 2 to nearly 30000. Cross-correlation analysis between S_t_ and B_t_ was also conducted, which was shown in Table 1.

**Table 1:** Results of cross-correlation analysis between daily original blogs (B_t_) and daily sales of Sony camera (S_t_).

Lags(i)	Direction	
	B_t-i_-> S_t_	S_t-i_->B_t_
1	*	-
2	-	-
3	-	-
4	-	-
5	*	-
6	*	-

B_t-i_-> S_t_ indicated forecasting S_t_ with B_t-i_, and S_t-i_-> B_t_ indicated forecasting B_t_ with S_t-i_, i indicated lags (daily).

*: Cross-correlation analysis was significant (p<0.01).

From Fig 1, we did not find the relationship between B_t_ and S_t_ intuitively, mainly due to the wide range of B_t_. We speculated that there might be a cross-correlation between the two time series data and the result (Table 1) supported our conjecture. In the lags (days) of 1, 5, 6, B_t_ was significantly cross-correlated with S_t_.

### Exploring transformation functions for daily original blogs (Bt) and daily sales of Sony camera (St)

The first chosen transformation was moving average, since it would reduce drastic fluctuations of the two time series data (B_t_ and S_t_). Then, we tried many different transformation functions including moving average, logarithmtics and combination of them with different parameters as far as possible. We ultimately determined the following combination of transformation functions as it was the best one in all groups we tried with higher significance level and better forecasting lags. The final transforming functions were:

$$\begin{array}{l} \text{Public mood variable }X_{t}=\ln\frac{B_{t-2}+B_{t-1}+B_{t}}{3}\\ \text{Camera sales variable }Yt=\frac{S_{t-1}+S_{t}}{2} \end{array}$$

**Fig 2:**
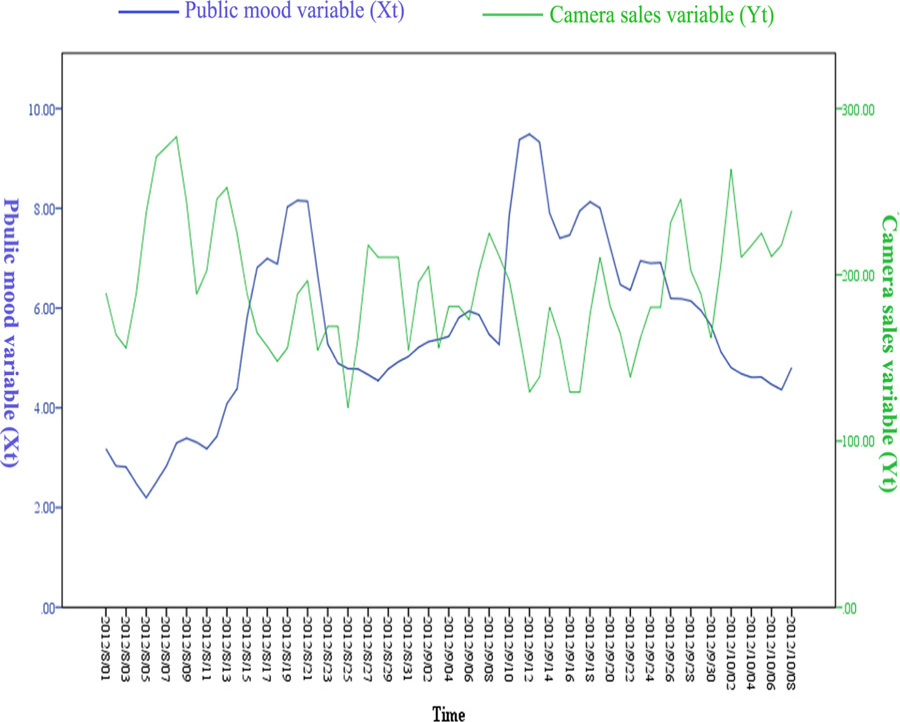
Trends of public mood variable (X_t_) and camera sales variable (Y_t_).

The trends of transformed time series data (X_t_ and Y_t_) could be seen in Fig 2. From the result, we could conjecture that there were negative correlations between X_t_ and Y_t_ intuitively. According to the curves of X_t_ and Y_t_, the 70-days periods could be further divided into six sub-periods: which are 2012/8/1 to 2012/8/13, 2012/8/14 to 2012/8/25, 2012/8/26 to 2012/9/10, 2012/9/11 to 2012/9/24, 2012/9/25 to 2012/9/30 and 2012/10/1 to 2012/10/8 respectively. During each sub-period, the negative cross-correlation between X_t_ and Y_t_ was more obvious.

### Cross-correlation test of public mood variable (Xt) and Camera sales variable (Yt)

**Fig 3:**
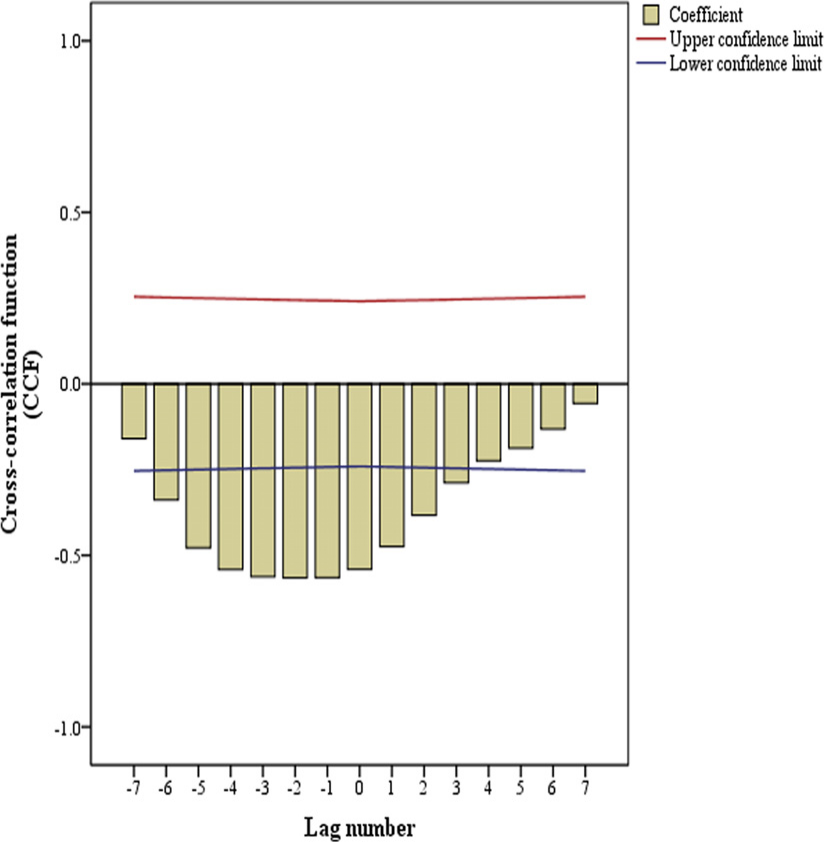
Cross-correlation coefficients of Y_t_ and X_t+i_.

In this section, Cross-correlation test of public mood variable (X_t_) and Camera sales variable (Y_t_) was conducted and the cross-correlation results could be seen in Fig 3.

In Fig 3, there were ten significant cross-correlation coefficients (p<0.05), which were X_t-6_ and Y_t_, X_t-5_ and Y_t_, X_t-4_ and Y_t_, X_t-3_ and Y_t_, X_t-2_ and Y_t_, X_t-1_ and Y_t_, X_t_ and Y_t_, X_t+1_ and Y_t_, X_t+2_ and Y_t_, X_t+3_ and Y_t_. Among these coefficients, the latter four were meaningless in predicting camera sales with public mood variable either because the forecasting direction was reversed (applying camera sales variable to predict public mood variable) or the advance lag is 0 (X_t_ and Y_t_). If we had applied the 6 preceding public mood variables (X_t-6_, X_t-5_, X_t-4_, X_t-3_, X_t-2_ and X_t-1_), there might be a high multicollinearity in regression model.

Thus, in order to determine what public mood variables should be chosen in final prediction models, we conducted regression analysis for each public mood variable (X_t-6_, X_t-5_, X_t-4_, X_t-3_, X_t-2_ and X_t-1_) and camera sales variable (Y_t_). The results were shown in Table 2.

**Table 2:** Regression analysis for each public mood variable (X_t-6_, X_t-5_, X_t-4_, X_t-3_, X_t-2_ and X_t-1_) and camera sales variable (Y_t_).

Model	Variables	Regression Coefficient	T	P	Adjusted R-square
1	X_t-1_	-11.844	-5.575	4.943×10^-7^	0.310
2	X_t-2_	-11.947	-5.605	4.571×10^-7^	0.315
3	X_t-3_	-12.027	-5.617	4.528×10^-7^	0.320
4	X_t-4_	-11.615	-5.294	1.611×10^-6^	0.297
5	X_t-5_	-10.383	-4.498	3.069×10^-5^	0.234
6	X_t-6_	-7.606	-3.046	0.003	0.118

independent variable: Y_t_

From the statistics, we could see that X_t-3_ had the highest t-value, the lowest p-value and the highest R-square, so the public mood variable (X_t-3_) would be included in final prediction models. Moreover, results of stepwise regression for all public mood variables (X_t-6_, X_t-5_, X_t-4_, X_t-3_, X_t-2_ and X_t-1_) and camera sales variable (Y_t_) also supported this choice:

$$Y_{t}=259.720-12.027X_{t-3}\;F=30.069,p<0.001;t=-5.484,p<0.001$$

In the above stepwise regression, other public mood variables (X_t-6_, X_t-5_, X_t-4_, X_t-2_ and X_t-1_) were all removed excluding X_t-3_.

Practically, when we use cross-correlation test to predict a time series data with another one, valuable information in the forecasted variable might be ignored. Therefore, autocorrelation of camera sales variable (Y_t_) would be studied in the next section.

### Autocorrelation and autoregression analyses of camera sales variable (Yt)

**Fig 4:**
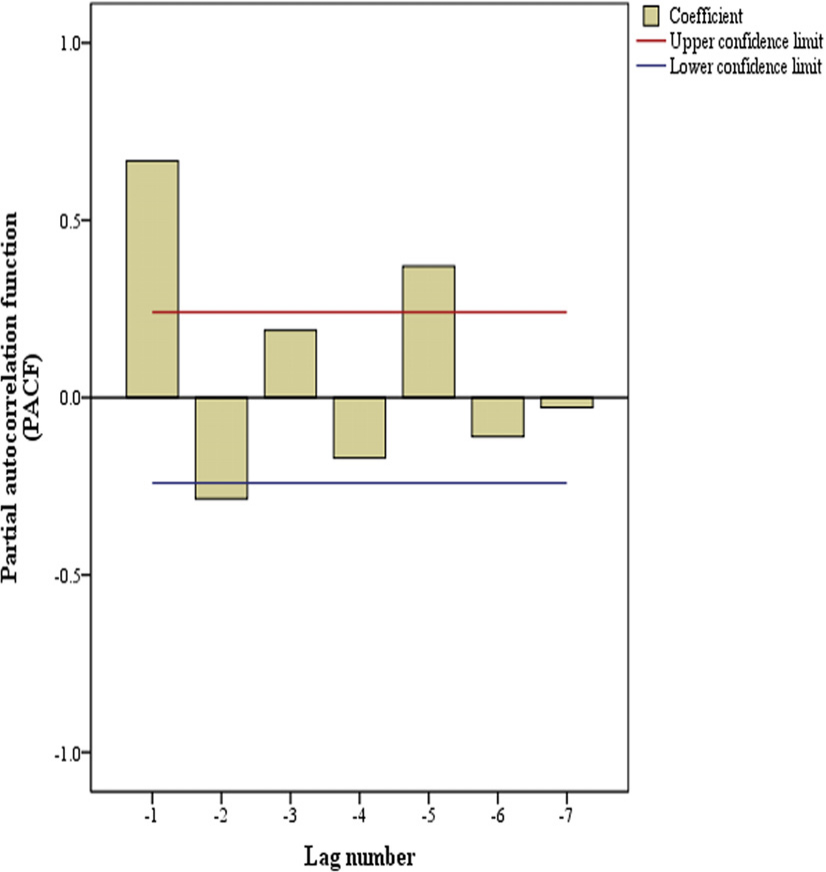
Partial autocorrelation results of camera sales variable (Y_t_).

Since partial autocorrelation coefficients correspond to autoregression models of time series data, we made autocorrelation analysis of Y_t_ (Fig 4).

From Fig 4, we could see that the camera sales variable (Y_t_) was partially autocorrelated at the advance lag (days) of 1, 2 and 5. Therefore, variables, Y_t-1_, Y_t-2_ and Y_t-5_, went into the stepwise regression with Y_t_ as dependent variable. There were two significant models just as follows:

$$\begin{array}{l} Y_{t}=62.580+0.675Y_{t-1},\;F=54.337,p<0.001;t=7.371,p<0.001\\ Y_{t}=81.283+0.876Y_{t-1}-0.299Y_{t-2},\;F=32.513,p<0.001;\;t_{1}=7.347,p<0.001;\;t_{2}=-2.505,p=0.015 \end{array}$$

In the results, variable Y_t-5_ was removed in the stepwise regression, because its p-value was more than 0.1. And Y_t-1_ and Y_t-2_ were used in autoregression. Thus, Y_t-1_ and Y_t-2_ were selected into final prediction models.

### Multiple regression models for Sony camera sales

Adopting the selected variables (X_t-3_, Y_t-1_ and Y_t-2_), we built prediction models for camera sales (Y_t_) applying stepwise regression (Table 3).

**Table 3:** Multiple regression models for camera sales (Y_t_).

Model	Variables	Regression Coefficient	T	P
1	Constant	63.481	3.495	0.001
	Y_t-1_	0.676	7.290	5.749×10^-10^
2	Constant	127.983	4.278	6.523×10^-5^
	Y_t-1_	0.515	4.779	1.096×10^-5^
	X_t-3_	-5.923	-2.648	0.010
3	constant	178.855	5.846	2.026×10^-7^
	Y_t-1_	0.737	6.390	2.412×10^-8^
	X_t-3_	-8.201	-3.844	2.877×10^-4^
	Y_t-2_	-0.422	-3.687	4.789×10^-4^

All of the three models were significant with p-values less than 1.00×10^-9^

independent variable: Y_t_

In Table 3, we could see that there were three significant models (all p-value<0.001) including different independent variables: Model 1 only included a sales variable (Y_t-1_), and Model 2 included a sales variable (Y_t-1_) and a public mood variable (X_t-3_). Model 3 included two sales variables (Y_t-2_ and Y_t-1_) and a public mood variable (X_t-3_). Equations of these models were as follows:

$$\begin{array}{l} Model1:Y_{t}=63.481+0.676Y_{t-1}\\ Model2:Y_{t}=127.983+0.515Y_{t-1}-5.923X_{t-3}\\ Model3:Y_{t}=178.855+0.737Y_{t-1}-8.201X_{t-3}-0.422Y_{t-2} \end{array}$$

Only Model 2 and Model 3 had a public mood variable (X_t-3_).

### Model estimation

In order to test the value of public mood variable in prediction of camera sales, we compared F statistic, R-square and prediction accuracy of Model 1 and Model 2. Forecasting accuracy was measured in terms of Mean Absolute Percentage Error (MAPE). The MAPE was defined as follows:

$$MAPE=\left(\frac{100}{n}\right)\sum_{t=1}^{n}\left|\frac{A_{t}-F_{t}}{A_{t}}\right|$$

Where A_t_ was the actual value and F_t_ was the predicted value at the time point t.

The results could be seen in Table 4, which showed that inclusion of the public mood variable (X_t-3_) (1) promoted significance (reduction of F-statistic, 53.146->32.575), (2) while increased R-square (0.454->0.508) and (3) reduced MAPE prediction error (12.70->11.35). Therefore, we would conclude that public mood could influence consumption choices, which was an appropriate indicator in forecast of sales.

**Table 4:** Estimation of Model 1 and Model 2.

Model	F-statistic	R-square	MAPE FOR Y_t_
Model 1: Y_t_ = 63.481+0.676Y_t-1_	53.146	0.454	12.70
Model 2: Y_t_ = 127.983+0.515Y_t-1_-5923X_t-3_	32.575	0.508	11.35

## Discussion

In previous studies, some scholars attempted to extract public mood indicators from a huge amount of online data (e.g. search engine and social media data) and studied their prediction validity in presidential election [1], economic operations [2], financial market indexes [3–5], and even the number of national suicides [6]. These researches [1–6] have demonstrated that it is feasible to extract public mood indicators from online data to make predictions. However, few previous researches have examined relationship, specially forecasting relationship between public mood and consuming choices at society level.

Concerned with the “Diaoyu Island” event, this study extracted Chinese public mood information toward Japan and Japanese products from social media, and then analyzed the cross-correlation between the public mood variable and sales variable applying suitable functions. Finally, three prediction models for Sony camera sales (Y_t_) were built, with public mood information and advance sales as independent variables. The results showed that: (1) the public mood variable could be significantly cross-correlated to the sale variable of a particular product and this correlativity could be used to build prediction models; (2) adding public mood variable in prediction models would promote the significance (reducing F-statistic), increase R-square and reduce MAPE prediction error in the prediction models. These results indicated that public mood was significantly associated with consumption choices and might be of value in sales forecasting for particular products. This study was the extension and supplement of previous data mining researches of online big data.

The main contributions of this study are as follows: 1) the present study paid attention to the “Diaoyu Island Event” between China and Japan, and empirically studied the influence of public mood on related consumption, which had not been studied by previous researches; 2) beyond correlation between public mood and related consumption, this study found that public mood might be valuable in forecasting sales of particular products; 3) the current paper discussed the approach of applying variable transformation to probe the correlativity between time series data, which might be a new way to analyze online big data in the future.

## Supporting Information

S1 Dataset Data of daily original blogs (Bt) and daily sales of Sony camera (St). (XLSX)
